# The Inhibitory Effect of Validamycin A on *Aspergillus flavus*

**DOI:** 10.1155/2020/3972415

**Published:** 2020-06-27

**Authors:** Napasawan Plabutong, Supanuch Ekronarongchai, Nattarika Niwetbowornchai, Steven W. Edwards, Sita Virakul, Direkrit Chiewchengchol, Arsa Thammahong

**Affiliations:** ^1^Medical Microbiology, Interdisciplinary Program, Graduate School, Chulalongkorn University, Bangkok, Thailand; ^2^Antimicrobial Resistance and Stewardship Research Unit, Department of Microbiology, Faculty of Medicine, Chulalongkorn University, Bangkok, Thailand; ^3^Institute of Integrative Biology, University of Liverpool, Liverpool, UK; ^4^Department of Microbiology, Faculty of Science, Chulalongkorn University, Bangkok, Thailand; ^5^Translational Research in Inflammation and Immunology Research Unit, Department of Microbiology, Faculty of Medicine, Chulalongkorn University, Bangkok, Thailand

## Abstract

*Aspergillus flavus* is one of the most common isolates from patients with fungal infections. *Aspergillus* infection is usually treated with antifungal agents, but side effects of these agents are common. Trehalase is an essential enzyme involved in fungal metabolism, and the trehalase inhibitor, validamycin A, has been used to prevent fungal infections in agricultural products. In this study, we observed that validamycin A significantly increased trehalose levels in *A. flavus* conidia and delayed germination, including decreased fungal adherence. In addition, validamycin A and amphotericin B showed a combinatorial effect on *A. flavus* ATCC204304 and clinical isolates with high minimum inhibitory concentrations (MICs) of amphotericin B using checkerboard assays. We observed that validamycin A and amphotericin B had a synergistic effect on *A. flavus* strains resistant to amphotericin B. The MICs in the combination of validamycin A and amphotericin B were at 0.125 *μ*g/mL and 2 *μ*g/mL, respectively. The FICI of validamycin A and amphotericin B of these clinical isolates was about 0.25–0.28 with synergistic effects. No drug cytotoxicity was observed in human bronchial epithelial cells treated with validamycin A using LDH-cytotoxicity assays. In conclusion, this study demonstrated that validamycin A inhibited the growth of *A. flavus* and delayed conidial germination. Furthermore, the combined effect of validamycin A with amphotericin B increased *A. flavus* killing, without significant cytotoxicity to human bronchial epithelial cells. We propose that validamycin A could potentially be used *in vivo* as an alternative treatment for *A. flavus* infections.

## 1. Introduction

*Aspergillus flavus* is a fungus commonly found in the environment, and when it contaminates food, it produces aflatoxins, which are associated with increased risk of developing liver cancer in humans [1, 2]. Moreover, *A. flavus* is an infectious fungus and can colonize organs leading to conditions such as keratitis, cutaneous infections, sinusitis, and invasive pulmonary aspergillosis [3–5]. Knowledge and understanding of the epidemiology and pathogenesis of *A. flavus* infection in humans are still very limited as there are only a few reports on *A. flavus* in comparison to other *Aspergillus* species [6]. For example, it has been reported that *A. flavus* is a common cause of cutaneous infections and sinusitis in India [4, 5].

Initial treatment of *Aspergillus* invasive infections (invasive aspergillosis) begins with antifungal agents, particularly azoles. Voriconazole is a drug of choice in patients with aspergillosis [7, 8], but serious adverse reactions have been reported in many studies, such as transient visual disturbances, hepatotoxicity, tachyarrhythmias, and QTc interval prolongations [8]. Amphotericin B is a fungicidal polyene agent, which is an alternative, relatively cheap treatment for aspergillosis [7, 8], but it also has serious side effects (e.g., nephrotoxicity) [9]. Owing to socioeconomic status of patients and availability of this agent, the use of amphotericin B as a treatment against aspergillosis is very common in developing countries, including Thailand [10–12]. Unfortunately, recent studies have demonstrated increasing incidence of *A. flavus* clinical isolates with resistance to amphotericin B [13, 14].

Although patients with aspergillosis are treated with standard antifungal therapy as mentioned, evidence shows that the morbidity and mortality rates in patients with these infections are still high (up to 80%) [15]. Therefore, the discovery of novel antifungal agents with fewer side effects is crucial for treatment of aspergillosis. Many studies have reported virulence factors and metabolic pathways that are specific to this fungus, and these could potentially be new targets for the development of antifungal agents [16, 17]. For example, trehalose is a disaccharide that is only found in bacteria, plants, insects, and invertebrates. It is composed of two glucose molecules conjugated with *α*, *α*-1, 1-glycosidic linkage, and serves as an energy source, particularly when fungi are exposed to environmental stresses such as cold, heat, and desiccation [18–20].

**Figure 1:**
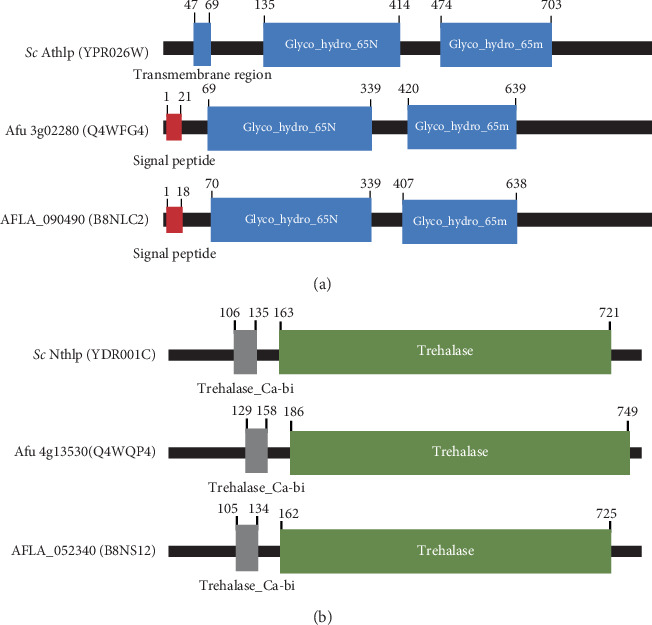
*Aspergillus flavus* possesses trehalase homologs. (a) Percentages of identity and similarity of *Sc*Ath1p (YPR026W) : AFLA_090490 (B8NLC2) and Afu3g02280 (Q4WFG4) : AFLA_090490 (B8NLC2) from BLASTp analyses are 29% identity, 46% similarity and 68% identity, 81% similarity, respectively. *Sc*Ath1p, *Saccharomyces cerevisiae* acid trehalase protein; Afu, *Aspergillus fumigatus*; AFLA, *Aspergillus flavus*; glycosyl hydrolase family 65 (Glyco_hydro_65N; Glyco_hydro_65m) (adapted from SMART analyses (http://smart.embl-heidelberg.de/)).(b) Percentages of identity and similarity of *Sc*Nth1p (YDR001C) : AFLA_052438 (B8NS12) and Afu4g13530 (Q4WQP4) : AFLA_052438 (B8NS12) from BLASTp analyses are 55% identity, 69% similarity and 81% identity, 88% similarity, respectively. *Sc*Nth1p, *Saccharomyces cerevisiae* neutral trehalase protein; *Afu*, *Aspergillus fumigatus*; AFLA, *Aspergillus flavus*; Trehalase_Ca-bi: neutral trehalase calcium-binding domain; trehalase: trehalose hydrolysis domain (adapted from SMART analyses (http://smart.embl-heidelberg.de/)).

There are three different enzymes involved in the trehalose pathway: (a) trehalose-6-phosphate synthase (Tps1p), (b) trehalose-6-phosphate phosphatase (Tps2p), and (c) trehalase (Figure 1). Tps1p converts UDP-glucose and glucose 6-phosphate into trehalose-6-phosphate [20]. Tps2p enzyme removes phosphate from trehalose-6-phosphate to form trehalose. These enzymes in the trehalose pathway are essential for the growth of *Candida albicans*, *Cryptococcus neoformans*, and *Aspergillus fumigatus* [18, 21–23]. Trehalase hydrolyzes and degrades trehalose into two glucose molecules [24]. There are two types of trehalase found in *Saccharomyces cerevisiae* [25], which are neutral trehalase and acid trehalase (Figure 1). Neutral trehalase (Nth1p) is found in the cytosol and works at an optimum pH of 7.0 [24, 26], whereas acid trehalase (Ath1p) is a cell wall-linked enzyme and works at an optimum pH of 5.0 [27–29]. It has been reported that the trehalose pathway is involved in the pathogenesis of fungal infections in humans (e.g., *C. albicans, C. neoformans,* and *A. fumigatus)* [19,21–23,30–32].

In previous studies, it was demonstrated that *Rhizoctonia solani,* a rice fungal pathogen, was inhibited by the trehalase inhibitor, validamycin A [33–35]. Validamycin A was originally isolated from *Streptomyces hygroscopicus* var. *limoneus* [33, 36, 37], and it was shown that it inhibited branching of *R. solani* [33, 38]. Another study found that validamycin A delayed conidial production of *Fusarium culmorum* [38]. However, the effectiveness of validamycin A against human fungal pathogens and its toxicity on human cells are unknown. Here, we investigated the effects of validamycin A alone and in combination with amphotericin B on the growth of *A. flavus*, including the cytotoxicity of validamycin A to a human cell line.

## 2. Materials and Methods

### 2.1. Fungal Strains, Media, and Conditions

*A. flavus* ATCC 204304 was cultured on Sabouraud dextrose agar (SDA, Oxoid, Thermo Fisher Scientific) Petri-dish plates at 37°C for three days before harvesting *A. flavus* conidia using sterile distilled water with 0.01% Tween 80. In brief, 5 mL of sterile distilled water with 0.01% Tween 80 was utilized to harvest *A. flavus* conidia on SDA Petri-dish plates using cell scrapers. The mixture between distilled water with Tween 80 and *A. flavus* conidia was filtered using Miracloth. A number of conidia were counted from the filtrate using a hemocytometer. Then, 10^3^ conidia were inoculated into culture media [39], i.e., glucose peptone agar (peptone 10 g, glucose 20 g, agar 20 g, distilled water 1000 ml, and pH 6.8–7.0), trehalose peptone agar (peptone 10 g, trehalose 10 g, agar 20 g, distilled water 1000 ml, and pH 6.8–7.0), and peptone agar (peptone 10 g, agar 20 g, distilled water 1000 ml, and pH 6.8–7.0), incubated at 37°C for 2–5 days. The radial fungal growth was measured in three biological replicates.

*A. flavus* clinical isolates were obtained from the Mycology Laboratory, Department of Microbiology, Faculty of Medicine, Chulalongkorn University, and King Chulalongkorn Memorial Hospital during 2019. Patient characteristics were collected from medical records/charts. Patients with invasive aspergillosis (IA) were classified as proven, probable, and possible invasive aspergillosis according to EORTC/MSG criteria [40, 41].

### 2.2. Trehalose Measurements

Conidia of *A. flavus* ATCC 204304 from SDA treated with or without 1 *μ*g/mL validamycin A were collected at day 5 after incubation at 37°C. Trehalose levels of *A. flavus* conidia were measured, as previously described [42]. In brief, 2 × 10^8^ conidia in 500 uL distilled water with Tween 80 were boiled at 100^o^C for 20 min and centrifuged at 11,000 ×g for 10 min. The supernatant was collected for trehalose measurement (with biological triplicates) using the glucose oxidase assay protocol (Sigma; GAGO20). The reaction was measured at 490 nm using a spectrophotometer (Lambda 1050+ UV/Vis/NIR, PerkinElmer, USA).

### 2.3. Germination Assay

Conidia of *A. flavus* ATCC 204304 at 1 × 10^8^ cells were incubated in 10 mL Sabouraud dextrose broth at 37°C in an orbital shaker at 200 rpm. The cultured broth (500 *μ*L) was used for counting percentage of germlings. The germinated conidia are counted using a microscope. At each time point, 100 conidia were counted, and the number of germinated conidia was calculated as a percentage out of total 100 conidia [43]. Each strain was cultured up to 24 h at 37°C in three biological replicates [44].

### 2.4. XTT Assay

XTT assays (sodium 2,3-bis (2-methoxy-4-nitro-5-sulfophenyl)-5-[(phenylamino)-carbonyl]-2H-tetrazolium) were performed as described previously [45, 46]. In brief, 10^3^ conidia of *A. flavus* ATCC 204304 were incubated with different culture media with or without validamycin A in a 96-well plate at 37°C for 18 h. XTT solution (0.5 mg/mL in PBS) was added into each well and incubated at 37°C for 15 min. The plate was centrifuged, and the supernatant was collected to measure the OD at 490 nm using a spectrophotometer (Lambda 1050+ UV/Vis/NIR, PerkinElmer, USA).

### 2.5. Crystal Violet Adherence Assay

10^5^ conidia per mL of *A. flavus* ATCC204304 were incubated in 100 *μ*L of Sabouraud dextrose broth in each well of plastic U-bottomed 96-well plates at 37°C for 24 h. After washing each well twice with sterile distilled water gently, 0.1% crystal violet was utilized to stain for 10 min. Sterile distilled water was then utilized to wash twice, and 100% ethanol was used to destain for 10 min. Supernatants were then measured at 600 nm using a spectrophotometer (Lambda 1050+ UV/Vis/NIR, PerkinElmer, USA) [47].

### 2.6. Broth Microdilution Assay and Checkerboard Assay

The CLSI broth microdilution M38 method was performed to observe the minimum inhibitory concentrations (MICs) of amphotericin B for *A. flavus* ATCC 204304 and clinical isolates [48]. The additive/synergistic effect of validamycin A and amphotericin B was identified using the checkerboard assays [49]. Fractional inhibitory concentration index (FICI) was calculated for each antifungal drug, in each combination used, with the following formula [49]:

(1) $$\begin{array}{c} \text{FICA}\left(\frac{{\text{MIC}}_{\text{A}}}{{\text{MIC}}_{\text{A}+\text{B}}}\right)+\text{FICB}\left(\frac{{\text{MIC}}_{\text{B}}}{{\text{MIC}}_{\text{A}+\text{B}}}\right)=\text{FICI.} \end{array}$$

FICI results were determined as follows: synergy: <0.5; additivity: 0.5–1; indifference: >1–4; and antagonism: >4.

### 2.7. Cell Line and Culture

BEAS-2B (human bronchial epithelial cell line) (ATCC® CRL9609™) was cultured with Bronchial Epithelial Cell Growth Basal Medium (BEBM) in tissue culture flasks coated with 0.01 mg/mL fibronectin, 0.03 mg/mL bovine collagen type I, and 0.01 mg/mL bovine serum albumin (BSA). The cells were incubated at 37°C in a humidified environment with 5% CO_2_ [50].

### 2.8. Cytotoxicity Assay

The cytotoxicity of validamycin A towards human epithelial cell lines was performed using a Lactate Dehydrogenase (LDH) Cytotoxicity Colorimetric Assay Kit II (BioVision Inc., CA, USA). In brief, 1 × 10^4^ BEAS-2B cells were incubated with 50 *μ*L of DMEM in a precoated 96-well plate, and then validamycin A was added at different concentrations (1 *μ*g/mL–1 mg/mL, final concentration). LDH reaction mixture was added, and the cells were incubated at 37°C for 30 min. LDH released from the cells was measured at 450 nm using a spectrophotometer. The percentage of cytotoxicity was calculated using the following formula:

(2) $$\begin{array}{c} \text{Cytotoxicity}\left(\%\right)=\;\frac{\left(\text{ test sample }-\text{ low control}\right)\times \;100}{\left(\text{high control }-\text{low control }\right)}. \end{array}$$

High control is cells with lysis buffer, while low control is cells alone as a background.

### 2.9. Statistical Analysis

All statistical analyses were conducted with Prism 8 software (GraphPad Software, Inc., San Diego, CA). Comparison between groups was performed with unpaired two-tailed Student's *t-*tests for two data groups and one-way ANOVA tests with post hoc Bonferroni's multiple comparison tests for more than two data groups. Error bars represent standard errors of the means. Significant differences were considered when *P* value < 0.05.

### 2.10. Ethical Statement

This study was approved by the Institutional Review Board (IRB no. 546/60), Faculty of Medicine, Chulalongkorn University, Bangkok, Thailand.

## 3. Results

### 3.1. Trehalase Homologs in *Aspergillus flavus*

To identify trehalase enzyme homologs in *A. flavus*, a BLASTp search was performed on *S. cerevisiae* and *A. fumigatus* and compared with *A. flavus*. The protein data from the FungiDB database and Simple Modular Architecture Research Tool (SMART) were used to compare putative protein domains among trehalase enzymes from *S. cerevisiae* (*Sc)*, *A. fumigatus* (*Afu)*, and *A. flavus* (*AFLA*) (database: https://fungidb.org and http://smart.embl-heidelberg.de).

The results showed that AFLA_090490 protein, containing one signal peptide at positions 1–18 and two O-glycosyl hydrolase domains (EC 3.2.1) at positions 70–339 and 407–638, was similar to acid trehalase of *S. cerevisiae* and *A. fumigatus* (Figure 1(a)). AFLA_052430 protein, containing a neutral trehalase calcium-binding domain at positions 105–134 and an O-glycosyl hydrolase domain (EC 3.2.1) at positions 162–725, was similar to neutral trehalase of *S. cerevisiae* and *A. fumigatus* (Figure 1(b)). Our findings suggest that *A. flavus* has both acid and neutral trehalases, as seen in *S. cerevisiae* and *A. fumigatus.*

**Figure 2:**
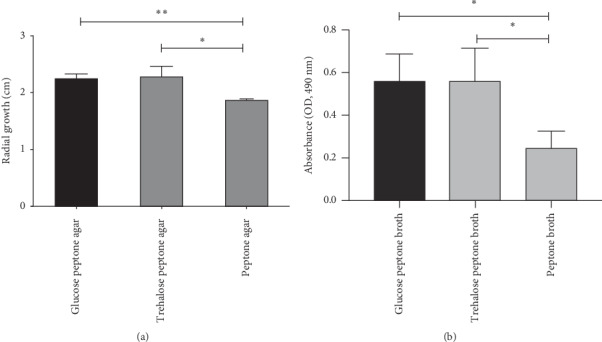
*Aspergillus flavus* utilizes trehalose as a sole carbon source similar to glucose. (a) *Aspergillus flavus* ATCC 204304 was incubated at 37°C on glucose peptone, trehalose peptone, and peptone alone media. The radial growth of these fungal growths was measured on the second day of incubation. Data are presented as means ± SE from three biological replicates. ^*∗*^*P* value < 0.05; ^*∗∗*^*P* value < 0.01 (one-way ANOVA with post hoc Bonferroni's test). (b) *Aspergillus flavus* ATCC 204304 was incubated at 37°C on glucose peptone, trehalose peptone, and peptone alone liquid media for 24 hours, and viability tests using XTT assays were performed. Data are presented as means ± SE from three biological replicates. ^*∗*^*P* value < 0.05 (one-way ANOVA with post hoc Bonferroni's test).

Next, we investigated the ability of *A. flavus* to utilize trehalose as a sole carbon source. The result showed that growth and viability of *A. flavus* on glucose peptone media and trehalose peptone media were similar (Figures 2(a) and 2(b)). This finding supports the idea that *A. flavus* utilizes trehalose as a sole carbon source and implies that it degrades extracellular trehalose into glucose for its growth.

### 3.2. Growth Inhibition and Decreased Fungal Adherence of *Aspergillus flavus* by Validamycin A

**Figure 3:**
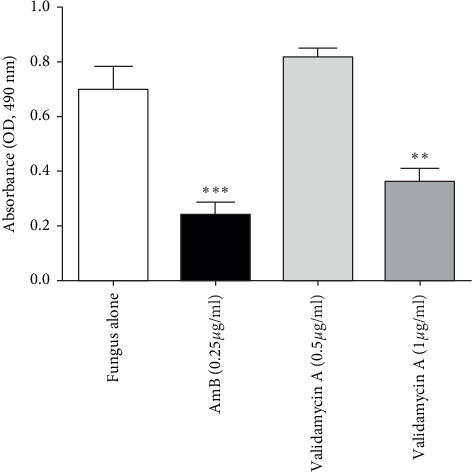
Validamycin A inhibits the growth of *Aspergillus flavus*. *Aspergillus flavus* ATCC204304 was cultured at 37°C in RPMI media in a 24-well plate for 18 hours. Fungal viability was measured by XTT assays at 490 nm. Amp, amphotericin B at 0.25 *μ*g/mL. Data are presented as means ± SE from three biological replicates. ^*∗∗*^*P* value < 0.01; ^*∗∗∗*^*P* value < 0.001 (one-way ANOVA with post hoc Bonferroni's test compared to fungus alone).

To observe the inhibitory effect of validamycin A on *A. flavus* ATCC204304, broth microdilution and XTT assays were performed. The results showed that the minimal inhibition concentration (MIC) of validamycin A against *A. flavus* was 1 *μ*g/mL (Table 1), and the viability of *A. flavus* ATCC204304 after validamycin A treatment at this concentration was significantly decreased when compared with 0.5 *μ*g/mL of validamycin A, 0.25 *μ*g/mL of amphotericin B, and the control group (Figure 3).

**Figure 4:**
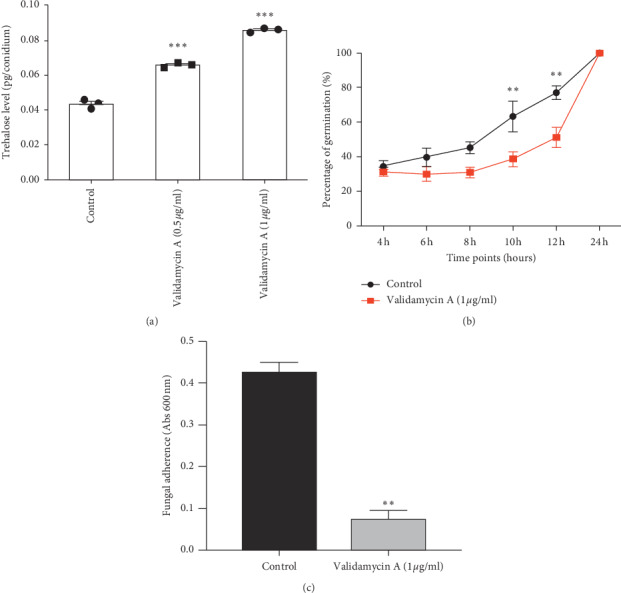
Validamycin A increases trehalose levels in *Aspergillus flavus* conidia with delayed conidial germination and decreased fungal adherence. (a) *Aspergillus flavus* ATCC 204304 was cultured at 37°C on Sabouraud dextrose agar for five days with or without 1 *μ*g/mL validamycin A. Trehalose assays were performed to measure trehalose levels in the conidia using glucose oxidase assays. Data are presented as means ± SE from three biological replicates. ^*∗∗∗*^*P* value < 0.001 (unpaired two-tailed Student's *t*-test compared with the control). (b) *Aspergillus flavus* ATCC 204304 was cultured at 37°C in Sabouraud dextrose broth with or without 1 *μ*g/mL validamycin A in an orbital shaker at 200 rpm. Conidial germination at each time point was counted and calculated. Data are presented as means ± SE from three biological replicates. ^*∗∗*^*P* value < 0.01 (unpaired two-tailed Student's *t*-test compared with the control). (c) *Aspergillus flavus* ATCC 204304 was cultured at 37°C in Sabouraud dextrose broth with or without 1 *μ*g/mL validamycin A in 96-well plates for 24 hours, and crystal violet adherence assays were performed. Data are presented as means ± SE from three biological replicates. ^*∗∗*^*P* value < 0.01 (unpaired two-tailed Student's *t*-test compared with the control).

Next, *A. flavus* ATCC204304 was cultured and treated with or without 0.5 and 1 *μ*g/mL of validamycin A, and trehalose levels in the conidia were measured. The results demonstrated that conidia collected from *A. flavus* treated with validamycin A showed significantly higher levels of trehalose than the control (untreated) group, suggesting that validamycin A inhibited trehalase enzymes in the conidia of *A. flavus* (Figure 4(a)). In addition, the rate of conidial germination was investigated in *A. flavus* conidia treated with 1 *μ*g/mL of validamycin A. The results showed that validamycin A significantly delayed conidial germination of *A. flavus* ATCC204304 particularly at 10 and 12 h (Figure 4(b)). These data suggest that validamycin A delays conidial germination of *A. flavus* via inhibition of trehalase enzymes.

To observe the effect of validamycin A on exopolysaccharides of *A. flavus*, the crystal violet adherence assays were performed. We observed that 1 *μ*g/mL of validamycin A decreased the adherence property of *A. flavus* ATCC204304 (Figure 4(c)). These data suggest that validamycin A affects the fungal adherence of *A. flavus*.

### 3.3. Synergistic Effects of Validamycin A and Amphotericin B on *Aspergillus flavus* Clinical Isolates

Antifungal susceptibility tests of *A. flavus* ATCC204304 were performed according to the CLSI broth microdilution method (CLSI M38, 2017). The results demonstrated that the MIC of validamycin A and amphotericin B alone against *A. flavus* ATCC204304 was 1 and 4 *μ*g/mL, respectively (Table 1). Furthermore, the fractional inhibitory concentration index (FICI) was 0.625 with the concentrations of validamycin A and amphotericin B at 0.125 *μ*g/mL and 2 *μ*g/mL, respectively (Table 1). This finding suggests that validamycin A and amphotericin B have an additive effect on *A. flavus* ATCC204304.

To confirm the combinative effects of validamycin A and amphotericin B, *A. flavus* clinical isolates (*n* = 3) with high MICs of amphotericin B (>4 *μ*g/mL) (Table 1) were chosen to perform checkerboard assays. Interestingly, the FICI was 0.25–0.28, suggesting a synergistic effect between these two drugs on these clinical isolates (Table 1).

### 3.4. No Cytotoxicity of Validamycin A to Human Bronchial Epithelial Cells

**Figure 5:**
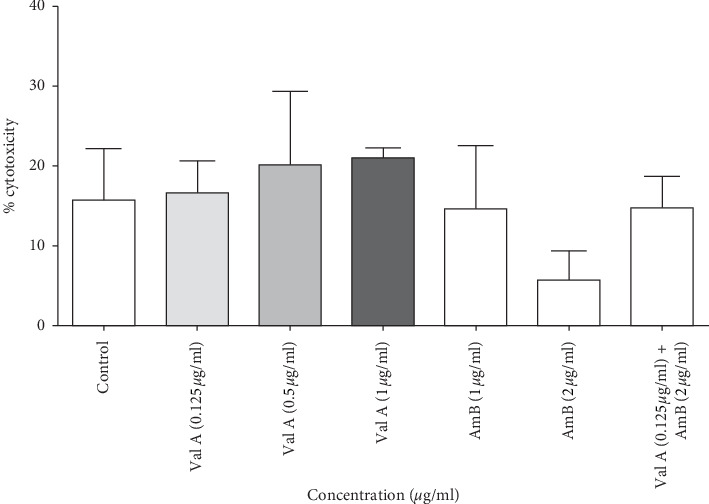
Validamycin A and the combination of validamycin A and amphotericin B have no cytotoxic effect on human bronchial epithelial cells. The cytotoxicity test was performed to observe the toxicity of validamycin A and amphotericin B on BEAS-2B cells using Lactate Dehydrogenase (LDH) Cytotoxicity Colorimetric Assay Kit II. Cell cultures were incubated at 37°C in a humidified environment containing 95% air and 5% CO_2_. After 24 hours, LDH reaction mixture was added (25 *μ*l) and incubated at 37°C for 30 minutes. Then, ODs were measured at 450 nm using a spectrophotometer. Data are presented as means ± SE from three biological replicates. No significant difference was observed (one-way ANOVA with post hoc Bonferroni's test).

Human bronchial epithelial cells, BEAS-2B, were treated with or without validamycin A including amphotericin B at different concentrations. The results demonstrated that 0.125, 0.5, and 1 *μ*g/mL of validamycin A, 1 and 2 *μ*g/mL of amphotericin B, and a combination of these two drug concentrations of 0.125 *μ*g/mL of validamycin A and 2 *μ*g/mL of amphotericin B showed no significant cytotoxicity to human bronchial epithelial cells (Figure 5).

## 4. Discussion

The trehalose pathway is a major mechanism for growth and metabolism of many fungi; however, the presence of trehalase enzymes in many of these fungi is still unknown [19, 21–23, 30–32]. Validamycin A is a trehalase enzyme inhibitor produced by *Streptomyces hygroscopicus* and is used for fungal inhibition in plants and insects [33, 36, 37, 51, 52]. From many previous reports, in plants and insects, the effect of validamycin A is to inhibit trehalase activity in their cells [53–56]. In a rice fungal pathogen, *Rhizoctonia solani*, validamycin A was shown to inhibit trehalase activity but not cellulase, pectinase, chitinase, amylase, or glucosidases [57]. Additionally, validamycin A also inhibited the growth of *Rhizoctonia solani* and *Fusarium culmorum* [33, 38]. However, there are only few studies demonstrating the effects of validamycin A on human fungal pathogens [58]. From our study, we observed that a human fungal pathogen, *A. flavus,* had two trehalase enzymes that shared similar conserved domains and possessed high similarity and identity to *Saccharomyces cerevisiae* and *Aspergillus fumigatus* (Figures 1(a) and 1(b)), including *Rhizoctonia solani* and *Candida albicans* (Figures S1(a)and S1(b)). Therefore, we hypothesize that validamycin A may inhibit trehalase enzyme activity in *A. flavus* similar to previous reports [33, 38, 57].

In this study, we investigated the presence of trehalase enzymes and the effect of the trehalase inhibitor, validamycin A, on the growth of a common pathogenic fungus in humans*, A. flavus*. The results showed that *A. flavus* possesses trehalase homologs and grows on trehalose peptone media, similar to growth on glucose peptone media (Figures 2(a) and 2(b)). These findings imply that *A. flavus* utilizes trehalase enzymes to degrade trehalose for use as a carbon source and energy. In addition, we observed inhibitory effects of validamycin A on the growth of *A. flavus* (Figure 3). This finding suggests that trehalase activity is required for *A. flavus* growth. However, direct evidence, such as genetic approaches (e.g., generating trehalase gene-deletion mutants) to support the importance of trehalase, is needed to confirm this observation.

In a previous study, it was found that validamycin A increased trehalose levels in a pathogenic fungus, *C. albicans* [58]. This result is similar to our findings that showed an increase in trehalose levels of *A. flavus* conidia after validamycin A treatment (Figure 4(a)). However, further trehalase activity assay using high-performance liquid chromatography (HPLC) is also necessary to confirm the effect of validamycin A against trehalase enzymes in *A. flavus*. As the trehalose pathway is crucial in the early stages of conidial germination [18, 19, 47, 59], we further investigated the effect of validamycin A on conidial germination of *A. flavus*. Expectedly, validamycin A significantly delayed conidial germination of *A. flavus* (Figure 4(b)). Therefore, these observations suggest that the inhibition of trehalase enzymes depletes the source of energy and the growth for *A. flavus*. Nonetheless, we observed that conidial germination, in the presence of validamycin A, was not different from the untreated group at 24-hour incubation. This result suggests that *A. flavus* could probably increase conidial germination by alternative pathways following trehalase inhibition (e.g., mannitol pathway) [60, 61]. A wide variety of different media is still necessary to further investigate the trehalose phenotypes in *A. flavus.*

In addition, this study further investigated the combinative effect between validamycin A and amphotericin B on *A. flavus* ATCC204304, which is a standard strain for the antifungal susceptibility test. The result demonstrated that these two drugs showed an additive effect on growth inhibition of *A. flavus.* Interestingly, the combination of these drugs had a synergistic effect on *A. flavus* clinical isolates with high MICs of amphotericin B. Although the cutoff value of MIC for amphotericin B resistance in *A. flavus* was unknown, Barchiesi et al. suggested that MIC of amphotericin *B* ≥ 2 *μ*g/mL should be considered as a resistant strain [48, 62].

Trehalose pathway is clearly associated with cell wall components, including chitin and beta-glucan, as shown in many previous reports [18, 19, 42, 47]. Disturbance in substrates of trehalose or enzymes or proteins associated with the trehalose pathway in *Aspergillus fumigatus* would lead to changes in the cell wall components and structure [18, 19, 42, 47]. Furthermore, trehalose level and proteins associated with the trehalose pathway may affect exopolysaccharide galactosaminogalactans (GAGs), which are important for fungal adherence and biofilm formation, as shown in *A. fumigatus* previous reports [42, 47]. In this study, we also observed that validamycin A decreased fungal adherence (Figure 4(c)). These data imply that the structure or components of exopolysaccharide GAGs may be affected by validamycin A.

Besides, trehalase enzymes in many eukaryotic organisms may play important roles in carbon metabolism, chitin biosynthesis, and stress tolerance, i.e., sucrose and trehalose homeostasis in *Arabidopsis thaliana* and *Phaseolus vulgaris*, regulation of chitin biosynthesis in insects, and carbon partitioning in many plants [63–70]. Therefore, we hypothesize that inhibition of the trehalase enzyme via validamycin A may change the structure and components of the fungal cell wall and exopolysaccharide through changes in the carbon metabolism of *A. flavus* leading to increased permeability and synergistic effects of amphotericin B against *A. flavus* in the presence of validamycin A. However, further studies of cell wall/GAG structures via the electron microscope and cell wall/GAG components through HPLC, including RNA sequencing and metabolomic analyses, are necessary to decipher the effect of validamycin A on *A. flavus* [18, 47].

Additionally, MICs of validamycin A in each *A. flavus* clinical isolate were varied. This variation of MICS of validamycin A in these clinical isolates is probably due to the difference in the cell wall/GAG structure and components of each strain (e.g., glucan or chitin), as a previous study showed that amphotericin B-resistant *A. flavus* contained higher (1,3)-*β*-D-glucan in their cell wall than the sensitive strains [71]. Furthermore, previous studies suggest that some clinical isolates of *A. fumigatus* had different phenotypes including cell wall components and virulence [72, 73].

We further characterized these clinical isolates and observed that the growth rate and conidial trehalose levels showed no difference from *A. flavus* ATCC204304 (Figures S2(a) and S2(b)). However, these isolates possessed different fungal adherence properties (Figure S2(c)). Different exopolysaccharide components and/or structure of these isolates may lead to decreased permeability of amphotericin B and validamycin A into the fungal cell membrane and cytoplasm affecting MICs in each clinical isolate. Nonetheless, the cell wall/GAG structure and components of these clinical isolates need to be further studied. Moreover, more clinical isolates and animal models are also necessary to confirm synergistic effects between validamycin A and amphotericin B.

Cytotoxicity of validamycin A was tested in our study, and the result demonstrated that validamycin A at concentrations showing synergistic effects on *A. flavus* had no cytotoxicity on human bronchial epithelial cells (Figure 5). Nevertheless, different human cell lines together with different concentrations of validamycin A and amphotericin B are still needed to be further investigated for the cytotoxicity. In addition, *in vivo* studies are required as acute toxicity was found in rodents at very high doses of validamycin A (https://pubchem.ncbi.nlm.nih.gov/compound/Validamycin-A). For future *in vivo* survival studies, different concentrations of validamycin A, i.e., 0.125 and 1 *μ*g/mL with or without the combination of amphotericin B, and different routes of administration, e.g., oral gavage, intraperitoneal route, or intravenous route, are necessary to be further investigated.

In conclusion, this study demonstrated that validamycin A delayed conidial germination and inhibited the growth of *A. flavus*. Moreover, a combination between validamycin A and amphotericin B showed a synergistic effect on amphotericin B-resistant *A. flavus* clinical isolates. The cytotoxicity of validamycin A to human bronchial epithelial cells was not observed in our study. Therefore, we propose that validamycin A could potentially be used as adjunctive therapy in patients with *A. flavus* infection, particularly those who are infected with amphotericin B-resistant strains.

## Figures and Tables

**Table 1:** Minimum inhibitory concentrations (MICs) of validamycin A alone, amphotericin B alone, or validamycin A in combination with amphotericin B on *Aspergillus flavus* ATCC204304 and *Aspergillus flavus* from clinical isolates. The table also contains patient characteristics, i.e., specimen source, diagnosis, and underlying disease, including the fractional inhibitory concentration index (FICI) and the interpretation of FICI (interpretation: (A) additive; (S) synergistic).

*A. flavus* strains	Specimen	Diagnosis (EORTC criteria)	Underlying disease	MICs of the single agent (*μ*g/mL)		MICs of combined agents (*μ*g/mL)		FICI (*μ*g/mL)	Interpretation
				Validamycin A	Amphotericin B	Validamycin A	Amphotericin B		
*A. flavus* ATCC204304	Human sputum			1	4	0.125	2	0.625	A
*A. flavus* SI 1	Left sphenoid sinus	Invasive aspergillosis (probable invasive aspergillosis)	Diabetes, hypertension, and dyslipidemia	>128	8	0.125	2	<0.251	S
*A. flavus* SP 2	Sputum	Invasive pulmonary aspergillosis (possible invasive aspergillosis)	Hepatitis C virus cirrhosis	1	8	0.0312	2	0.281	S
*A. flavus* EN 3	Endotracheal aspiration	Invasive pulmonary aspergillosis (probable invasive aspergillosis)	Acute lymphoblastic leukemia	>128	8	0.0039	2	<0.250	S

## Data Availability

All data used to support the findings of this study are included within the article, and the raw data for each figure are available from the corresponding author upon request.

## Supplementary Materials

http://smart.embl-heidelberg.de/http://smart.embl-heidelberg.de/ Supplementary Materials Figure S1: *Aspergillus flavus* shares similar trehalase enzymes with *Rhizoctonia solani* and *Candida albicans.* (a) Percentages of identity and similarity of AFLA_090490 (B8NLC2) : *R. solani* AGM46811.1 (R4VJL2) and AFLA_090490 (B8NLC2) : *C. albicans* SC5314 acid trehalase (Q5AAU5) from BLASTp analyses are 31% identity, 47% similarity and 32% identity, 48% similarity, respectively. AFLA, *Aspergillus flavus*; glycosyl hydrolase family 65 (Glyco_hydro_65N; Glyco_hydro_65m); trehalase: trehalose hydrolysis domain (adapted from SMART analyses). (b) Percentages of identity and similarity of AFLA_052438 (B8NS12) : *R. solani* AGM46812.1 (R4VM92) and AFLA_052438 (B8NS12) : *C. albicans* P78042 neutral trehalase from BLASTp analyses are 55% identity, 70% similarity and 55% identity, 71% similarity, respectively. AFLA, *Aspergillus flavus*; Trehalase_Ca-bi, neutral trehalase calcium-binding domain; trehalase: trehalose hydrolysis domain (adapted from SMART analyses). Figure S2: different *Aspergillus flavus* isolates show no difference in the radial growth rate and conidial trehalose levels but possess different fungal adherence properties. (a) *Aspergillus flavus* ATCC 204304 and three clinical isolates were incubated at 37°C on glucose media. The radial growth of these fungal growths was measured on the third day of incubation. Data are presented as means ± SE from three biological replicates. No significant difference was observed (one-way ANOVA with post hoc Bonferroni's test). (b) *Aspergillus flavus* ATCC-204304 and three clinical isolates were cultured at 37°C on Sabouraud dextrose agar for five days with or without 1 *μ*g/mL validamycin A. Trehalose assays were performed to measure trehalose levels in the conidia using glucose oxidase assays. Data are presented as means ± SE from three biological replicates. No significant difference was observed (one-way ANOVA with post hoc Bonferroni's test). (c) *Aspergillus flavus* ATCC 204304 and three clinical isolates were cultured at 37°C in Sabouraud dextrose broth with or without 1 *μ*g/mL validamycin A in 96-well plates for 24 hours, and crystal violet adherence assays were performed. Data are presented as means ± SE from three biological replicates. ^*∗*^*P* value < 0.05; ^*∗∗*^*P* value < 0.01 (one-way ANOVA with post hoc Bonferroni's test compared to the ATCC-204304 strain).
