# Supplementary material for: The Inhibitory Effect of Validamycin A on Aspergillus flavus
Source: Int J Microbiol. 2020 Jun 27;2020:3972415. doi: 10.1155/2020/3972415 (PMC7336217; doi:10.1155/2020/3972415)
Supplement: http://smart.embl-heidelberg.de/http://smart.embl-heidelberg.de/ Supplementary Materials — Figure S1: Aspergillus flavus shares similar trehalase enzymes with Rhizoctonia solani and Candida albicans. (a) Percentages of identity and similarity of AFLA_090490 (B8NLC2) : R. solani AGM46811.1 (R4VJL2) and AFLA_090490 (B8NLC2) : C. albicans SC5314 acid trehalase (Q5AAU5) from BLASTp analyses are 31% identity, 47% similarity and 32% identity, 48% similarity, respectively. AFLA, Aspergillus flavus; glycosyl hydrolase family 65 (Glyco_hydro_65N; Glyco_hydro_65m); trehalase: trehalose hydrolysis domain (adapted from SMART analyses). (b) Percentages of identity and similarity of AFLA_052438 (B8NS12) : R. solani AGM46812.1 (R4VM92) and AFLA_052438 (B8NS12) : C. albicans P78042 neutral trehalase from BLASTp analyses are 55% identity, 70% similarity and 55% identity, 71% similarity, respectively. AFLA, Aspergillus flavus; Trehalase_Ca-bi, neutral trehalase calcium-binding domain; trehalase: trehalose hydrolysis domain (adapted from SMART analyses). Figure S2: different Aspergillus flavus isolates show no difference in the radial growth rate and conidial trehalose levels but possess different fungal adherence properties. (a) Aspergillus flavus ATCC 204304 and three clinical isolates were incubated at 37°C on glucose media. The radial growth of these fungal growths was measured on the third day of incubation. Data are presented as means ± SE from three biological replicates. No significant difference was observed (one-way ANOVA with post hoc Bonferroni's test). (b) Aspergillus flavus ATCC-204304 and three clinical isolates were cultured at 37°C on Sabouraud dextrose agar for five days with or without 1 μg/mL validamycin A. Trehalose assays were performed to measure trehalose levels in the conidia using glucose oxidase assays. Data are presented as means ± SE from three biological replicates. No significant difference was observed (one-way ANOVA with post hoc Bonferroni's test). (c) Aspergillus flavus ATCC 204304 and three clinical isolates were cultured at 37°C in Sabou [file 3972415.f1.docx]

**Supplementary materials**


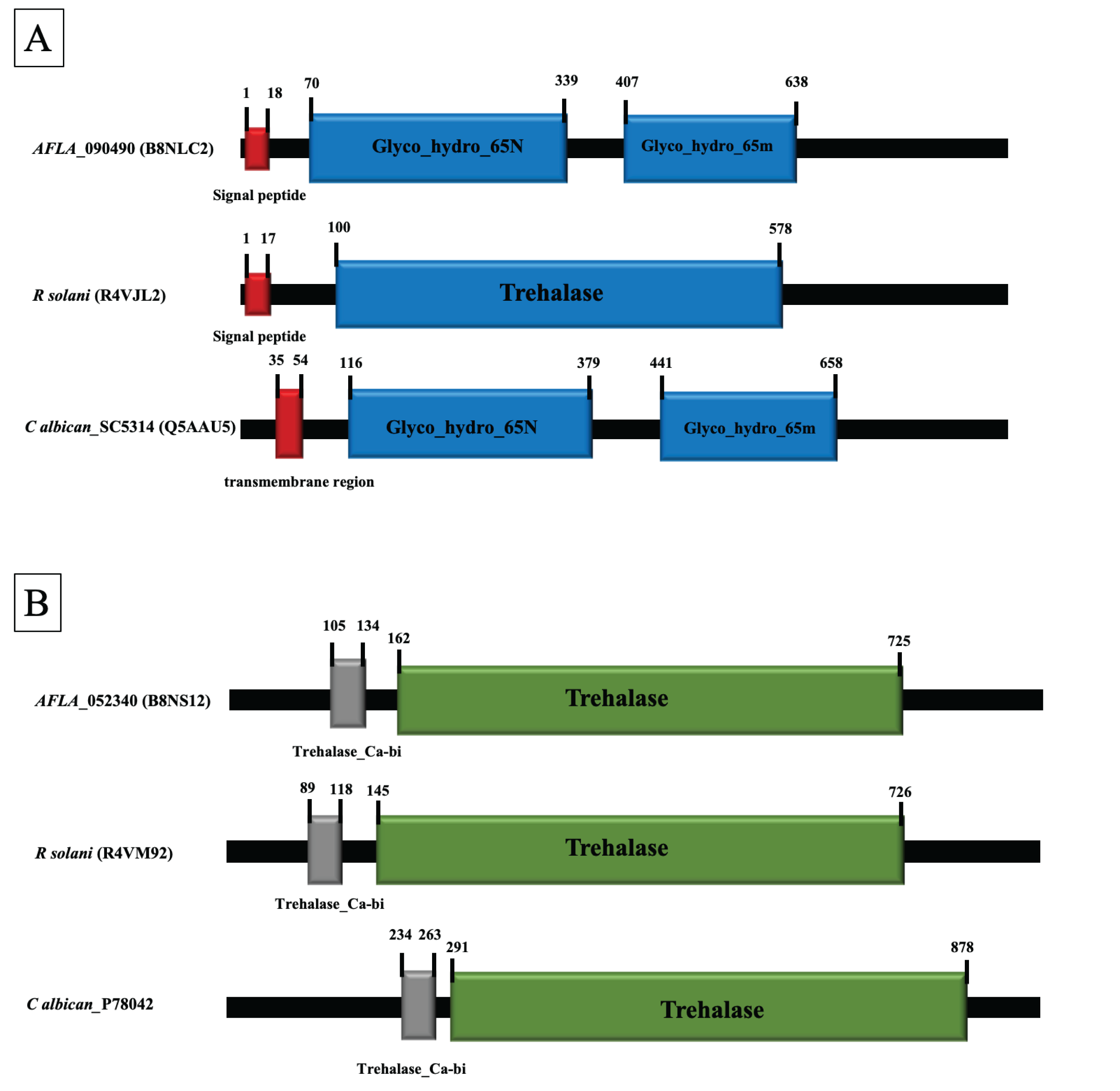


**Figure S1. *Aspergillus flavus* shares similar trehalase enzymes with *Rhizoctonia solani* and *Candida albicans****.* A) Percentages of identity and similarity of *AFLA_090490* (B8NLC2) : *R. solani* AGM46811.1 (R4VJL2) and *AFLA*_090490 (B8NLC2) : *C. albicans* SC5314 acid trehalase (Q5AAU5) from BLASTp analyses, are 31% identity, 47% similarity, and 32% identity, 48% similarity, respectively. *AFLA*: *Aspergillus flavus*; Glycosyl hydrolase family 65 (Glyco_hydro_65N; Glyco_hydro_65m); Trehalase: Trehalose hydrolysis domain. (Adapted from SMART analyses (<http://smart.embl-heidelberg.de/)>). B) Percentages of identity and similarity of *AFLA*_052438 (B8NS12) : *R. solani* AGM46812.1 (R4VM92) and *AFLA*_052438 (B8NS12) : *C. albicans* P78042 neutral trehalase from BLASTp analyses, are 55% identity, 70% similarity, and 55% identity, 71% similarity, respectively. *AFLA*: *Aspergillus flavus*; Trehalase_Ca-bi: Neutral trehalase calcium-binding domain; Trehalase: Trehalose hydrolysis domain. (Adapted from SMART analyses (<http://smart.embl-heidelberg.de/)>).


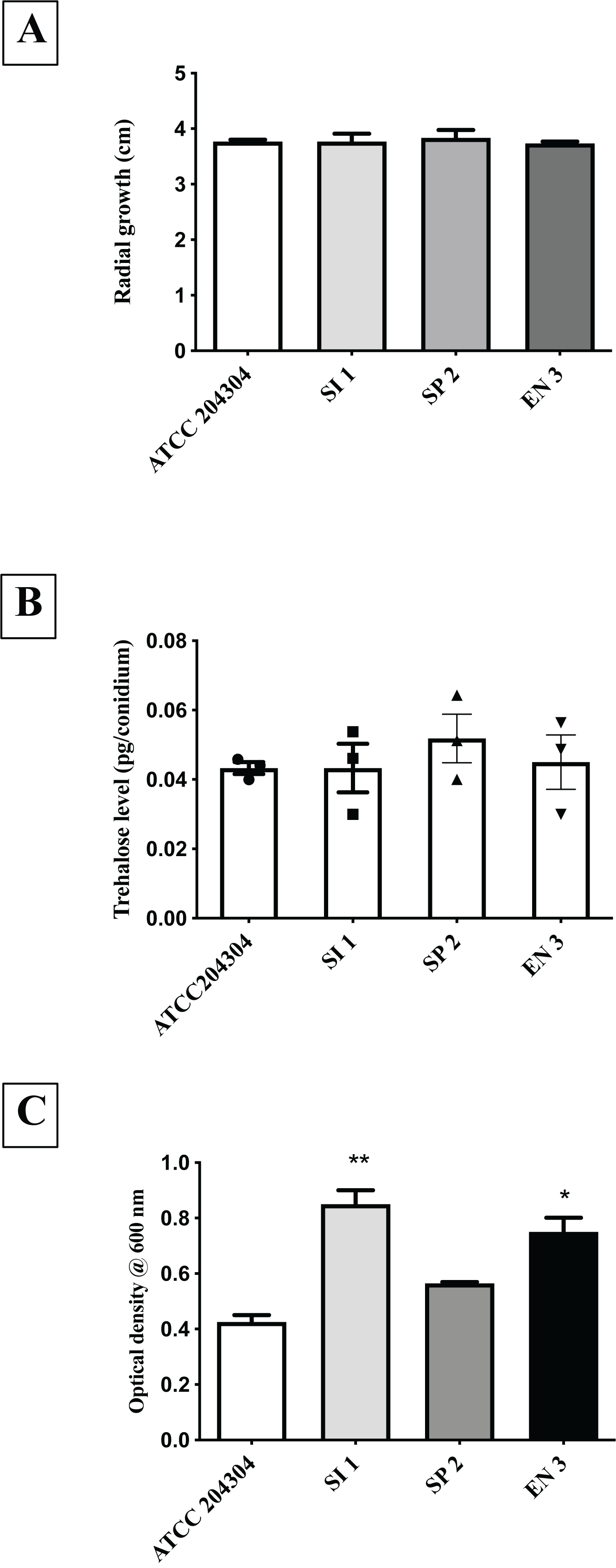


**Figure S2. Different *Aspergillus flavus* isolates show no difference in the radial growth rate and conidial trehalose levels, but possess different fungal adherence property.** A) *Aspergillus flavus* ATCC 204304 and three clinical isolates were incubated at 37⁰C on glucose media. The radial growth of these fungal growths was measured on the third day of incubation. Data are presented as means ± SE from three biological replicates. No significant difference was observed (one-way ANOVA with post-hoc Bonferroni's test). B) *Aspergillus flavus* ATCC 204304 and three clinical isolates were cultured at 37⁰C on Sabouraud dextrose agar for five days with or without 1 µg/mL validamycin A. Trehalose assays were performed to measure trehalose levels in the conidia using glucose oxidase assays. Data are presented as means ± SE from three biological replicates. No significant difference was observed (one-way ANOVA with post-hoc Bonferroni's test). C) *Aspergillus flavus* ATCC 204304 and three clinical isolates were cultured at 37⁰C in Sabouraud dextrose broth with or without 1 µg/mL validamycin A in 96-well plates for 24 hours and the crystal violet adherence assays were performed. Data are presented as means ± SE from three biological replicates. *, *P*-value < 0.05; **, *P*-value < 0.01 (one-way ANOVA with post-hoc Bonferroni's test compared to ATCC204304 strain).
